# A generalisable linkage pipeline (GLADIS) to facilitate research for the public good

**DOI:** 10.23889/ijpds.v8i2.2219

**Published:** 2023-09-14

**Authors:** Vellanki Pratibha, Mary Cleaton

**Affiliations:** 1 University of Manchester, Manchester, United Kingdom; 2 Office for National Statistics, Newport, United Kingdom

## Objectives

The Integrated Data Service (IDS) is a new cross-government service that facilitates research for the public good. Key to its success are Integrated Data Assets (IDAs): de-identified, grouped datasets that are joinable on an artificial ID and themed on a given topic. The Demographic Index (DI) comprises five linked administrative datasets. We are developing a generalisable method that will link administrative and survey datasets to the DI via a customisable, reproducible pipeline, to produce IDAs.

## Method

The method focuses on the traditional methodologies of deterministic and probabilistic data linkage and uses the Splink implementation of the Fellegi-Sunter method for probabilistic matching. The pipeline will include a tool for quality-assurance (QA) via clerical review.

We are researching a generalisable implementation of Splink, deriving the method’s control parameters using the results of the deterministic matching. Additionally, we are researching application of Locality Sensitive Hashing (LSH), a dimensionality-reduction method suggested to improve computational efficiency, for blocking. This is especially important due to the large size of the datasets involved.

## Results

We plan to produce linked datasets with three quality levels – prioritising precision, balancing precision and recall and prioritising recall. As the datasets are always linked to the DI, the DI’s artificial ID can be used as a ‘spine’ to bring them together as assets (IDAs).

Initially, the method will be used on the 2021 England and Wales Census. Despite not including clerical matching in the method (except for quality-assurance), we anticipate a high precision and recall due to the quality of the Census and the number of linkage variables available. Thereafter, we plan for user testing with other datasets, including the Labour Market Survey.

## Conclusion

Our generalisable linkage pipeline for the DI will, through its IDA outputs, facilitate research for the public good. This research will directly impact government policy and responses to national health emergencies, including Covid-19, and support government priorities such as Levelling Up and the transition towards Net Zero.

